# Interactive effect of branch source-sink ratio and leaf aging on photosynthesis in pistachio

**DOI:** 10.3389/fpls.2023.1194177

**Published:** 2023-08-02

**Authors:** Giulia Marino, Paula Guzmán-Delgado, Emily Santos, Jaclyn A. Adaskaveg, Bárbara Blanco-Ulate, Louise Ferguson, Maciej A. Zwieniecki, Eduardo Fernández-Suela

**Affiliations:** ^1^ Department of Plant Sciences, University of California, Davis, Davis, CA, United States; ^2^ Instituto Madrileño de Investigación y Desarrollo Rural, Agrario y Alimentario, Madrid, Spain

**Keywords:** light response curve, senescence, non-structural carbohydrates, alternate bearing, thinning, defoliation formatted: default paragraph font, font: formatted: font: not italic

## Abstract

Tree source-sink ratio has a predominant and complex impact on tree performance and can affect multiple physiological processes including vegetative and reproductive growth, water and nutrient use, photosynthesis, and productivity. In this study, we manipulated the branch level source-sink ratio by reduction of photosynthetic activity (partial branch defoliation) or thinning branch fruit load early in the growing season (after fruit set) in pistachio (*Pistacia vera*) trees. We then characterized the leaf photosynthetic light response curves through leaf aging. In addition, we determined changes in leaf non-structural carbohydrates (NSC) and nitrogen (N) concentrations. In leaves with high source-sink ratios, there was a gradual decrease in maximum net photosynthetic rate (A_Nmax_) over the growing season, while in branches with low source-sink ratios, there was a sharp decline in A_Nmax_ in the first two weeks of August. Branches with high-sink showed an up-regulation (increase) in photosynthesis toward the end of July (at 1,500 growing degree days) during the period of rapid kernel growth rate and increased sink strength, with A_Nmax_ being about 7 μmol m^-1^ s^-1^ higher than in branches with low-sink. In August, low source-sink ratios precipitated leaf senescence, resulting in a drastic A_Nmax_ decline, from 25 to 8 μmol m^-1^ s^-1^ (70% drop in two weeks). This reduction was associated with the accumulation of NSC in the leaves from 20 to 30 mg g^-1^. The mechanisms of A_Nmax_ reduction differ between the two treatments. Lower photosynthetic rates of 8-10 μmol m^-1^ s^-1^ late in the season were associated with lower N levels in high-sink branches, suggesting N remobilization to the kernels. Lower photosynthesis late in the season was associated with lower respiration rates in low-source branches, indicating prioritization of assimilates to storage. These results can facilitate the adaptation of management practices to tree crop load changes in alternate bearing species.

## 1 Introduction

Maximizing agro-ecosystems’ carbon assimilation has focused the attention of researchers for many years. Increasing net primary production is key for the economies of wood and food production systems and it plays a major role in the global carbon cycle (Ryan et al., 1997; Evans, 2013). Multiple environmental factors can constrain the photosynthetic carbon gain of the plant or system. For example, drought stress can impose both stomatal and non-stomatal limitations to photosynthesis (Scalisi et al., 2020; Marino et al., 2021), high temperature can inhibit photosynthesis (Djanaguiraman et al., 2020) and promote respiration leading to carbon starvation (Murphy and Way, 2021), unbalanced tree nutrition can reduce net photosynthesis (Loustau et al., 1999; Sperling et al., 2019), and viruses can reduce mesophyll conductance to CO_2_ and Rubisco activity (Sampol et al., 2003).

In addition to these abiotic and biotic factors, the plant’s internal physiological and morphological factors can also strongly inhibit or enhance photosynthesis. Among these intrinsic factors affecting photosynthesis, leaf age, and sink-source balance are particularly important. A leaf’s potential photosynthetic performance depends significantly on its age. Photosynthesis is parabolic through the season, increasing with leaf expansion, reaching its maximum at full leaf development, and declining with senescence (Jurik, 1986; Warren and Adams, 2001; Xu et al., 2019). Reductions in the carboxylation rate, electron transport (Warren, 2006), nitrogen remobilization, decrease in the Rubisco to chlorophyll ratio (Warren and Adams, 2001) and chloroplast degradation (Camp et al., 1982; Wittenbach et al., 1982) accompany the final leaf decline.

The periods between leaf development and senescence represent a significant fraction of the leaf lifespan; hence, changes in the timing of these phenological stages can strongly modify leaf lifetime carbon assimilation (Wilson et al., 2001; Niinemets et al., 2012; Guzmán-Delgado et al., 2017). The proportion between source and sink organs within a branch or a tree can affect photosynthesis directly by enhancing or inhibiting it or indirectly by modifying leaf ontogeny. Researchers have contrasting views on whether the sources (organs that are the net producers of photoassimilates) or the sinks (organs that are the net importers of photoassimilates) have the main effect on photosynthesis and the most recent conclusion is that both, combined, should be considered (Evans, 2013).

Iglesias et al. (2002) girdled, partially thinned fruits, and defoliated citrus trees to modify their source-sink ratio. They found that leaf photosynthetic rates decreased after fruit thinning and increased after leaf removal. Andrade et al. (2019) demonstrated that reduced leaf photosynthetic rates in fruit-thinned nectarine trees was associated with lower stomatal conductance and sugar accumulation in leaves and not with impaired photochemical processes. In contrast, the increase in leaf photosynthetic rates in response to defoliation is often associated with a reduction in total non-structural carbohydrates (NSC) in leaves. However, Pinkard et al. (2011) found that, in *Eucalyptus globulus*, such an increase in photosynthesis in response to defoliation was linked to the degree of resource availability, with the largest increase being attained in source-limiting treatments and with no increase being observed in sink-limiting treatments. Genotype and environmental conditions can also affect the response of carbon assimilation to thinning and defoliation in maize (Abeledo et al., 2020). Source-sink ratio interacts with leaf age, accelerating or delaying leaf senescence (Paul and Foyer, 2001; Sekhon et al., 2012) and impacting orchard carbon assimilation potential at the season’s end (Marino et al., 2018b). Most studies agree on the central role of NSC hyperaccumulation and nitrogen remobilization in triggering early leaf senescence when source-sink ratios are manipulated (Christensen et al., 1981; Ceppi et al., 1987; Sekhon et al., 2012), but the exact mechanism of action is complex and still not clear.

**Figure 1:**
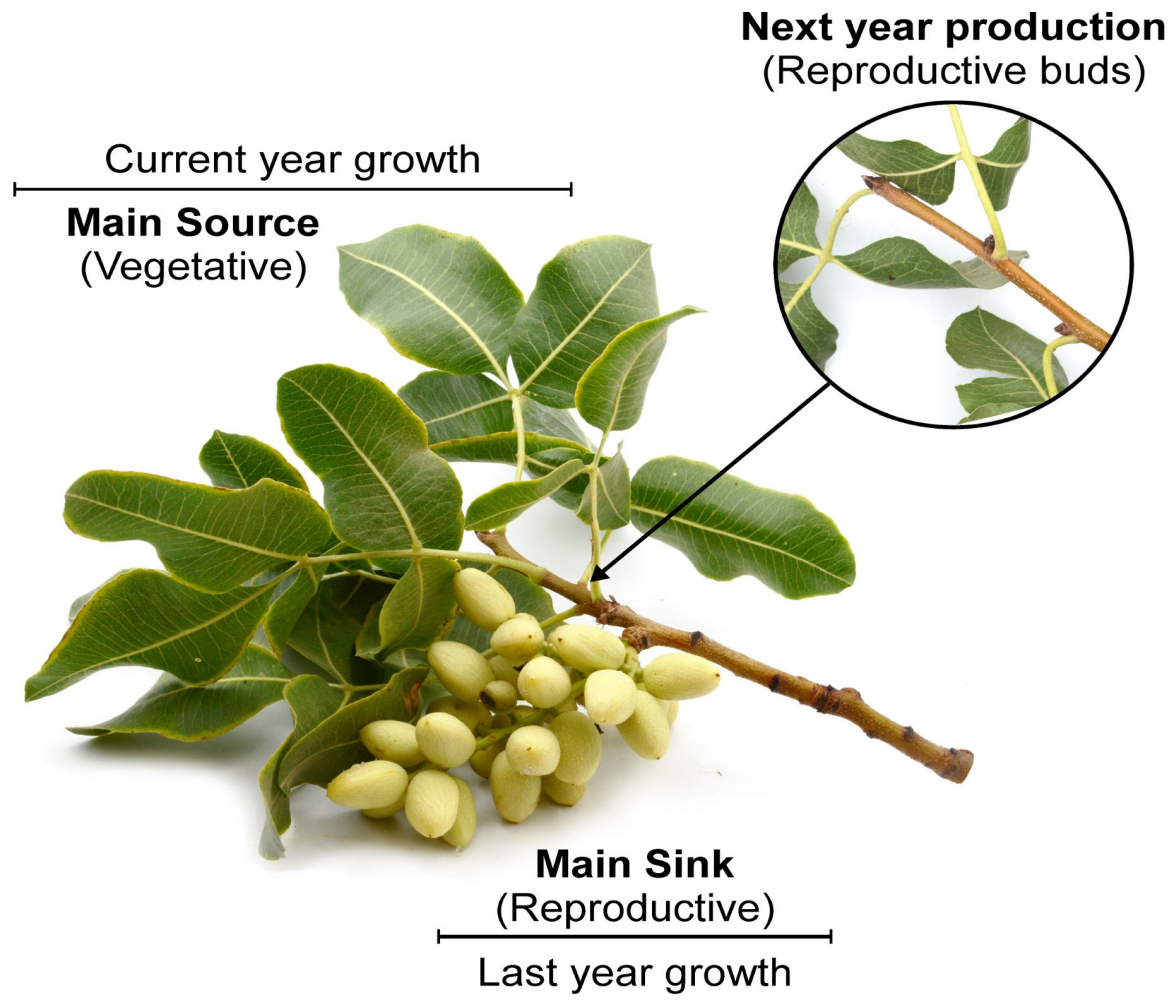
Schematic representation of a typical pistachio branch, with the main sink (the fruit cluster) located proximal to the main source (the leaves), on the previous and current year’s growth, respectively. The reproductive buds, representing the following year production, are in the leaf axils of the current year’s growth.

*Pistacia vera* has a strong apically dominant growth habit with the primary sink, the fruits, borne of the previous year’s growth, and the primary source, the leaves, borne of the current year’s growth (**Figure 1**). Pistachio trees have marked annual changes in the source-sink ratio within the branch that strongly affect productivity. A heavy fruit load will precipitate abscission of the reproductive buds and later cause early leaf senescence (Marino and Marra, 2019; Benny et al., 2020; Benny et al., 2022). The result in the following season within the individual branch is no fruit or alternate bearing, and less shoot growth due to decreased NSC reserves (Barone et al., 2014). These characteristics make pistachio trees perfect candidates to investigate the effect of manipulating source-sink relationships on leaf photosynthesis as their leaves age. Previous works in pistachio reported higher photosynthetic rates in bearing branches versus non-bearing branches only in the first part of the season (Vemmos, 1994, Baninasab et al., 2010; Sajjadinia et al., 2010). After July, a decline was observed in photosynthetic rates in bearing branches and trees that was associated with an early leaf senescence precipitated by the branch crop load. However, all these studies have been performed in rainfed or supplemental irrigated orchards, where water stress may have hindered the potential of branches to achieve higher photosynthetic rates, particularly later in the season when soil water resources are at their minimum. In addition, the branches or trees analyzed were always naturally bearing or no bearing fruits due to the alternate bearing phenomenon typical of this species. These branches or trees may have started the season with very different levels of resources, including starch (Crane and AI-Shalan, 1977) or nutrients such as N, P, Mg, K and Ca (Brown et al., 1995; Baninasab et al., 2007). For instance, in Vemmos’s study (1994) specific leaf mass was higher in fruiting than non-fruiting trees early in the season, when differences in photosynthetic rates were observed. According to this author, the earlier leaf emergence and maturation observed could have triggered these differences in photosynthesis. Finally, none of these studies have considered the influence of branch leaf area on photosynthesis.

In this work, we manipulated source-sink ratios in pistachio branches by partial thinning and defoliation and characterized leaf photosynthetic performance through the season as dictated by tree phenological and physiological changes. The information developed can be used to evaluate the potential impacts of resource competition on the leaf’s seasonal carbon gain potential.

## 2 Materials and methods

### 2.1 Experimental site

The study was conducted on a 10-year-old cv. Kerman on UCB1 rootstock commercial pistachio (*Pistacia vera* L.) orchard in Woodland, CA (latitude 38° 43’ 28” N, longitude -121° 48’ 38” W, elevation 23.58 meters). The soil was a Yolo silt loam. Trees were grown using standard commercial practices. Irrigation was managed by matching crop evapotranspiration (ETc) using reference ET from CIMIS station #226 and crop coefficient from Goldhamer (2005).

### 2.2 Meteorological data

Air temperature was monitored using two Onset HOBO Pendant ^®^ Data Loggers (UA-002-64) attached to two tree branches at opposite ends of the orchard. Additionally, meteorological data were acquired through a CIMIS weather station near the experimental site (CIMIS #226, 38° 40’ 22”, -121° 48’ 42”). Temperature data from the data loggers in the orchard was used to calculate accumulated heat Growing Degree Days (GDD) starting at full bloom using daily average temperature and the base temperature of 7 °C:


$$GDD=\left(\frac{t_{1}+\ldots +t_{n}}{n}\right)-7$$

The climatic data for the experimental location is displayed in **Table 1**. April and November were the coldest months, with temperatures ranging between 5-8 °C to 21-27 °C. The highest temperatures were recorded between May and August when temperatures reached a minimum of 15 °C and a maximum of 33-36 °C. Relative humidity ranged between 14-20% to 88-98%. Two rain events of 0.25 mm each were recorded in June and October.

**Table 1:** Climate data collected from the nearest CIMIS station #226 in Woodland, California, and calculated growing degree days (GDD, °C).

Sampling Date	GDD	Rain (mm)	Min T (°C)	Max T (°C)	Min RH (%)	Max RH (%)	Ψ_STEM_ (bars)
4/19/20	15	0	8.3	21.7	39	84	–
4/27/20	134	0	10.0	31.1	23	67	–
5/9/20	309	0	11.6	33.9	14	72	–
5/25/20	517	0	15.0	36.1	22	70	-10.5 ( ± 0.3)
6/19/20	934	0.25	13.9	35.5	20	68	-11.0 ( ± 0.5)
7/18/20	1450	0	15.0	35.0	–	–	-11.7 ( ± 0.7)
8/4/20	1775	0	15.5	33.3	35	85	-10.1 ( ± 0.9)
8/20/20	2088	0	15.0	32.2	27	78	-10.8 ( ± 0.7)
9/3/20	2324	0	14.4	33.9	34	88	-10.5 ( ± 0.8)
9/17/20	2577	0	12.2	32.8	31	98	-10.3 ( ± 0.4)
10/9/20	2904	0.25	12.2	27.8	–	–	–
11/4/20	3209	0	5.5	27.2	25	84	-12.8 ( ± 0.2)

The midday stem water potential (**Ψ_STEM_**, bars) for each measurement day is also presented.

### 2.3 Treatments

**Figure 2:**
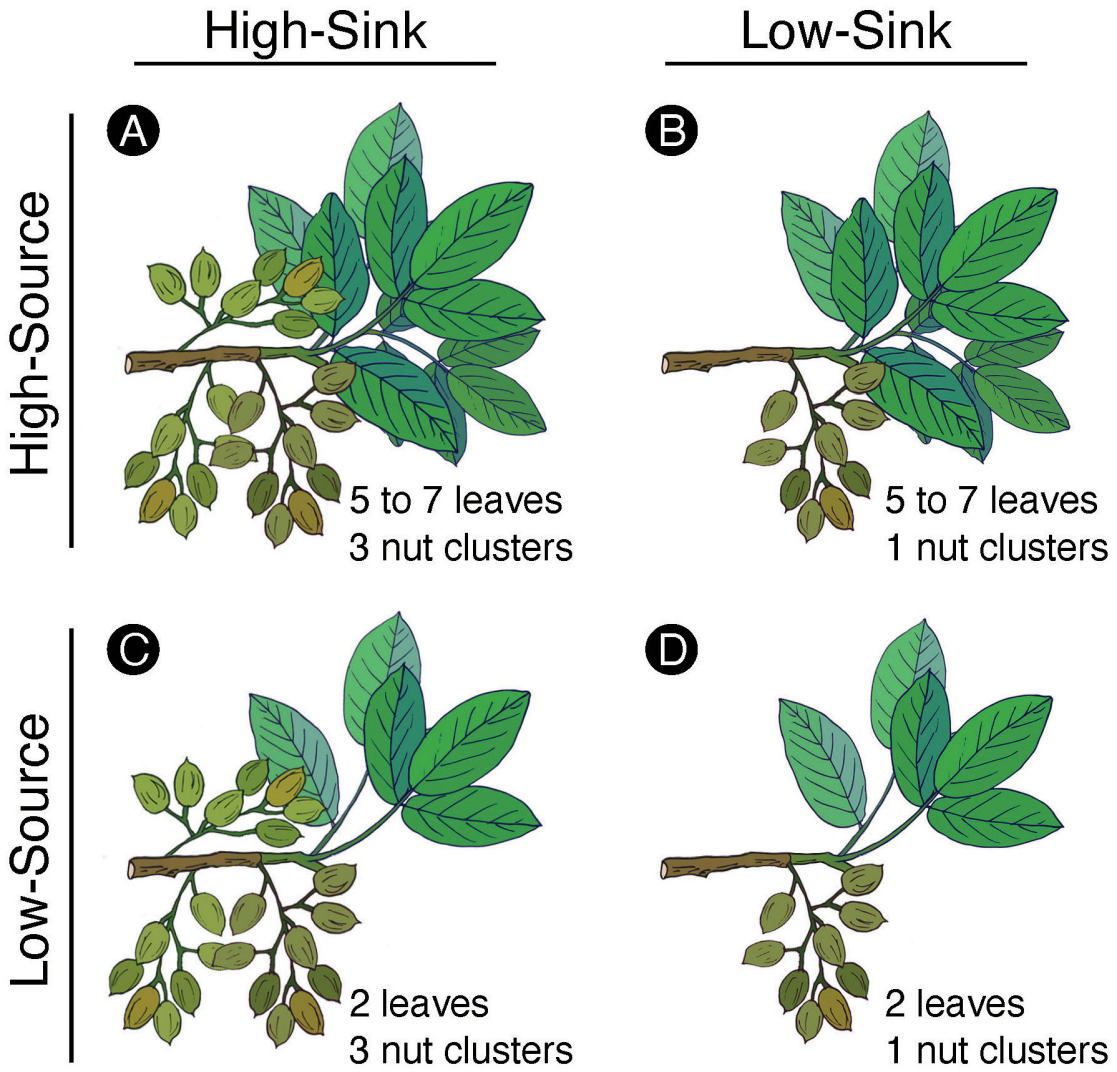
Schematic representation of the four combination treatments applied to the pistachio branches, with the ‘high-source:high-sink’ treatment **(A)** having naturally five to seven leaves and three clusters, the ‘high-source:low-sink’ treatment **(B)** having five to seven leaves and one cluster left after a manual thinning the ‘low-source:high-sink’ treatment **(C)** having two leaves left after being manually defoliated and naturally three clusters, and ‘low-source:low-sink’ treatment **(D)** having two leaves and one cluster after defoliation and thinning.

Manipulative treatments to alter branch sink-source balance were carried out in twenty homogenous trees randomly selected across five rows. Eight branches per tree, with at least three to four nut clusters and at least five leaves each, were chosen on the east and west sides of the canopy (four branches per side) on April 19th, right after the fruit set had occurred. At this time, the fruit load was manipulated in four of the eight branches selected on each tree by removing the nut clusters so that only one cluster remained on the branch. This manipulative thinning led to two treatments: ‘high-sink’ (branches with three clusters) and ‘low-sink’ (branches thinned with one cluster left). A total of 80 branches made up each fruit thinning treatment. On June 1st, when leaves were fully expanded, and fruit had completed the shell expansion, half of the ‘high-sink’ branches and half of the ‘low-sink’ branches were partially defoliated by detaching all but two leaves on the branch. This manipulative defoliation led to two additional treatments: ‘high-source’ (branches with 5 to 7 leaves) and ‘low-source’ (branches defoliated with two leaves). The combined manipulations resulted in four treatments made of 40 branches each (**Figure 2**): ‘high-source:high-sink’ (~five to seven leaves and three clusters), ‘low-source:high-sink’ (two leaves and three clusters), ‘high-source:low-sink’ (~five to seven leaves and one cluster), ‘low-source:low-sink’ (two leaves and one cluster). Each treatment was replicated twice per tree (once per each side of the tree). The defoliation treatment was applied slightly later in the season with respect to the fruit thinning treatment to avoid a too strong carbon deficit at the beginning of nut growth that could have promoted nut drop and impacted the branch fruit load.

### 2.4 Gas-exchange measurements

Once every two to three weeks, from the 27^th^ of April (15 GDD) until the 4^th^ of November (3209 GDD), the response curves of net photosynthesis (A_N_) at variable photosynthetic flux density (PPFD) were determined on the apical leaves of the selected branches. The response curves were performed between 11:00 and 13:00 h on three randomly chosen trees; one curve per treatment was performed in each tree. Measurements were taken using a portable photosynthesis system (Model LI-6800, LI-COR, Biogeosciences, Lincoln, NE, USA). Damaged or dried leaflets were excluded. Each measurement began with approximately 2-3 minutes of saturating light (1,500 μmol m ^−2^ s ^−1^) followed by six incremental reductions (1,200, 900, 600, 300, 150, and 0 μmol m ^−2^ s ^−1^) to produce photosynthesis versus incoming radiation (Q_in_) response curves. During the measurements, temperature and CO_2_ concentration inside the cuvette were maintained at constant levels (approximately 27°C and 380 ppm), and the airflow rate within the system was set at 400 µmols^–1^. Contemporaneously, stem water potential (Ψ_STEM_) was measured using a pressure chamber (PMS Instrument Co., Corvallis, OR) between 12:00 and 15:00 h, in the same trees where the light response curves were performed to ensure optimal tree water status. For Ψ_STEM_ measurements, a composite fully expanded and shaded leaf with three to five leaflets was selected from a non-bearing branch in the inner mid-canopy, covered with light-reflecting and moisture-impervious Mylar bags for at least 20 min, and then detached from the branch. A small piece of absorbent filter paper was placed on the exposed end of the petiole cut-off to determine the “endpoint” of actual xylem water emerging with applied pressure. Stem water potential (Ψ_STEM_) ranged between -10 and -12 bars (**Table 1**), which is considered a non-stress condition in pistachio (Memmi et al., 2016).

### 2.5 Biometric measurements

At the end of the experiment the tagged branches were sampled and brought to the lab to measure nut and leaf dry weight. Leaf area was also measured by taking a picture and subsequently analyzing it with Image J software (NIH, Bethesda, Maryland; Rasband, 2011). Source:sink ratio was estimated dividing the branch leaf area by the total branch nut dry weight (cm^2^ of leaf area per gram of nut dry matter).

### 2.6 Leaf analysis

At the end of July, when nut filling was complete, three branches per treatment were sampled every other week and brought to the lab. Leaflets were excised from the branches, washed with deionized water, oven-dried at 75°C for three days, and ground into a fine, homogeneous powder. The powder was then separated for NSC and nitrogen content analyses. Leaf NSC content was analyzed as per Leyva et al. (2008), with some modifications. For soluble carbohydrate or sugar extraction, 25 mg of sample were mixed with 1 mL 0.2 M sodium acetate buffer, pH 5.5, and incubated in a water bath at 70°C for 15 min. After centrifugation for 10 min at 21,000 g, the supernatant was diluted in bi-distilled water (1:20, v:v). Sugars were then quantified by adding 0.1% anthrone reagent dissolved in 98% sulfuric acid (m:v), incubating for 20 min at 100°C, cooling for 10 min, and reading absorbance at 620 nm in a spectrophotometer (Multiskan GO, Thermo Scientific). The remaining buffer and pellet were used for starch extraction. Samples were incubated at 100 °C for 10 min and then cooled for 20 min. Subsequently, 0.7 Uml^–1^ of amylase and 7 Uml^–1^ of amyloglucosidase were added, and the samples were stirred in a rotary incubator at 37°C for 4 h to digest starch. Samples were centrifuged at 21,000 g for 10 min, and the supernatant was diluted in distilled water. Sugars were quantified as described before. Starch concentration was determined by subtracting pre-starch digestion sugar content from total (pre- and post-starch digestion) sugar content. Two repetitions per sample were performed, and the results averaged. For the nitrogen content, 5 mg of ground sample was encapsulated whole into tin (Sn) capsules and sent to the UC Davis Stable Isotope Facility where they were combusted at 1000°C in a reactor packed with chromium oxide and silvered copper oxide. Following combustion, oxides were removed in a reduction reactor (reduced copper at 650°C). The helium carrier then flows through a water trap (magnesium perchlorate and phosphorous pentoxide). N_2_ is separated on a Carbosieve GC column (65°C, 65 mL/min) before being analyzed for ^15^N isotopes using a Elementar vario MICRO cube elemental analyzer (Elementar Analysensysteme GmbH, Langenselbold, Germany) interfaced to a Sercon Europa 20-20 isotope ratio mass spectrometer (Sercon Ltd., Cheshire, UK).

### 2.7 Data analysis

The non-rectangular hyperbola model proposed by Marshall and Biscoe (1980) was used to fit A_N_–PPFD response curves:


$$A_{\text{N}}=\frac{\text{αI }+\;A_{\text{Nmax }}-\;\sqrt{{\left(\text{αI }+\;A_{\text{Nmax}}\right)}^{2\;}-\;4\text{αI}A_{\text{Nmax}}\text{θ}}}{2\text{θ}}\;-\;R_{\text{d}}$$

where: A_N_ – leaf net assimilation (μmol m ^−2^ s ^−1^); α – apparent quantum yield (mol mol^–1^); A_Nmax_ – maximum photosynthetic capacity (μmol m ^−2^ s ^−1^); I – PPFD (m ^−2^ s ^−1^); θ – curvature factor [dimensionless]; R_D_ – dark respiration (μmol m ^−2^ s ^−1^).

The R package ‘photosynthesis’ was used to fit the equation and extrapolate the different parameters (Stinziano et al., 2020). Analysis of variance (ANOVA) was performed to compare the effects of treatment and time of the season on the photosynthetic parameters extrapolated from the curves using R (R Core Team, 2013). A two-way ANOVA was performed to study the effect of time and fruit load treatment on all the photosynthetic parameters. A three-way ANOVA was applied to a reduced dataset, with measured data starting June 1^st^, to compare the effect of the defoliation treatment, which had been applied only at that time of the season. The tree was included in the analysis as random effect. Differences were determined with the Tukey test, with statistical significance set at a p-value (p) <0.05.

## 3 Results

The manipulation treatments significantly affected the branch biometric parameters measured (**Table 2**), with the fruit thinning influencing significantly the dry weight of all the fruits in the branch and the defoliation influencing significantly the dry weight and the area of all the leaves in the branch. The branch source: sink ratio was influenced by both manipulative treatments (defruiting and defoliation) and their interaction (**Table 2**). The fruit thinning resulted in a ~70% reduction of sink weight per branch, from about 24 g in high-sink branches to about 8 g in low-sink branches (**Table 3**). The defoliation reduced leaf dry weight by 55% (from 7 to 3 g) and leaf surface area by 60% (490 to 200 cm^2^). These changes resulted in very different source:sink ratios. Removing only the leaves led to a source sink ratio of 8 (‘low-source:high-sink’ treatment); removing both leaves and fruits resulted in a source:sink ratio of 25 (‘low-source:low-sink’ treatment); eliminating the fruits led to a source:sink ratio of 104 (‘high-source:low-sink’ treatment); the non-manipulated treatment (‘high-source:high-sink’) had a source sink ratio of 21.

**Table 2:** Analysis of variance (ANOVA) testing the effects of source:sink manipulation on branch average dry weights (DW) of the fruits and of leaves (g), leaf area (LA, cm^2^), and actual source:sink ratio (cm^2^ g^-1^).

Factor	Fruits DW (g)	Leaves DW (g)	LA (cm^2^)	Source : Sink	
Sink	***	ns	ns	***	
Source	ns	***	***	***	
Sink x Source	ns	ns	ns	***	

ns, not significant.

*** p ≤ 0.001.

**Table 3:** Average dry weights (DW) of the fruits and of leaves (g), leaf area (LA, cm^2^), and actual source:sink ratio (cm^2^ g^-1^) per branch as a function of the treatments.

Source	Sink	Fruits DW (g)		Leaves DW (g)		LA (cm^2^)		Source : Sink	
Low	High	25.4 a		3.1a		203a		11c	
Low	Low	8.1b		3.1a		206a		38b	
High	High	23.3a		6.8b		487b		25bc	
High	Low	7.9b		7.5b		505b		104a	

For each parameter, different letters indicate significant differences among treatments based on a Tukey test (p ≤ 0.05).

**Figure 3:**
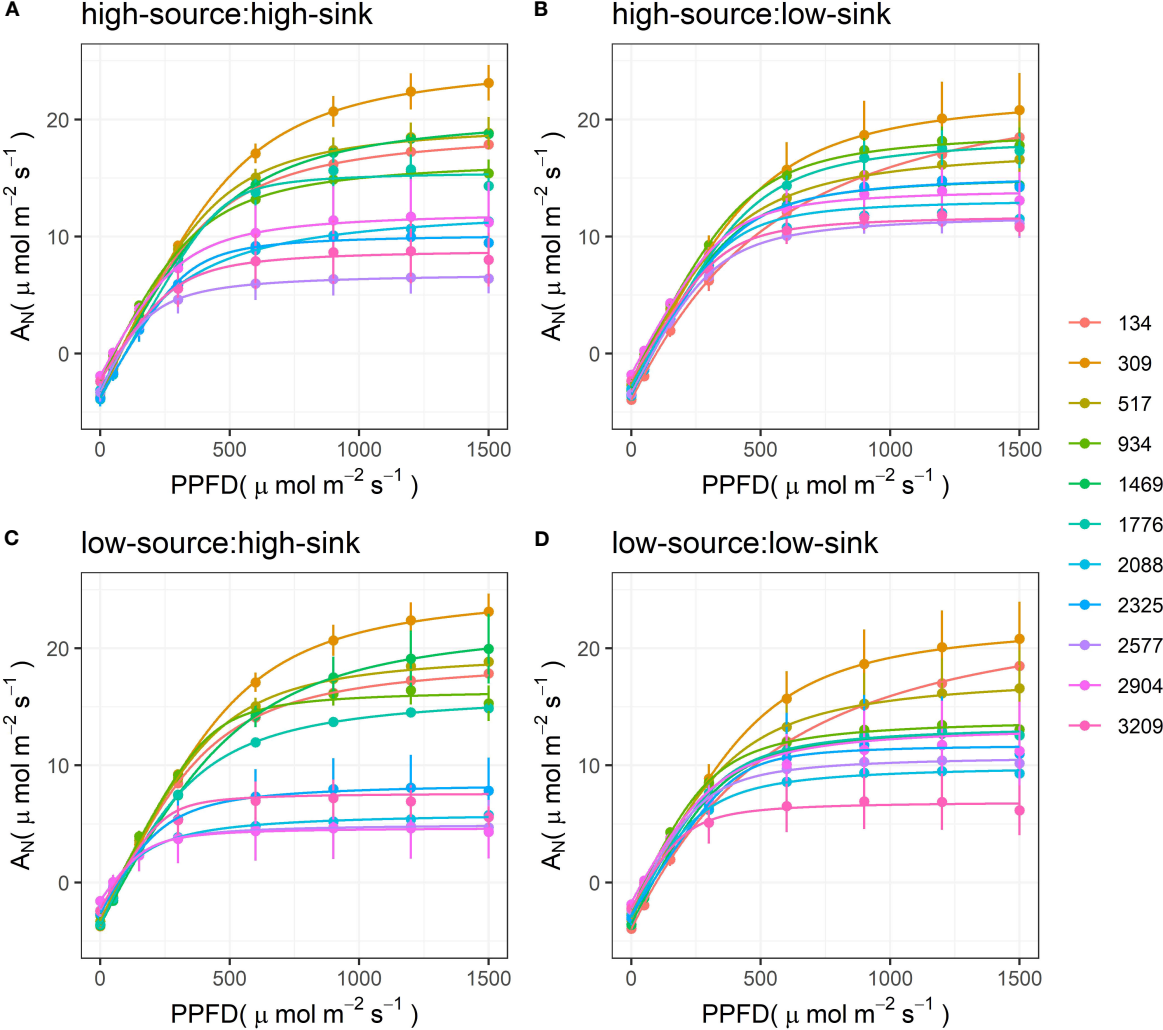
Response curves of net assimilation rate (A_N_) to photosynthetic photon flux density (PPFD) measured on pistachio leaves at various degrees of leaf development expressed as growing degree days in non-manipulated branches with high sink (3 clusters) and high source (~ 7 leaves) **(A)** and branches with artificially reduced sink **(B)**, source **(C)**, and both sink and source **(D)**. Symbols with error bars represent the measured data (n=3), and solid lines represent the modeled data using a non-rectangular hyperbola.

The light response curves of pistachio leaves varied through the season, with higher asymptotes (*A_N_*_max_) earlier, and lower asymptotes later (**Figure 3**). The main exception to this trend was the curve measured at 134 GDD at the end of April; it had a lower asymptote than the curves measured in the subsequent days over the different treatments and a slower, smoother increase in A_Nmax_ at low PPFD values. The low-sink treatments tended to have the most compact group of curves, with *A*_Nmax_ homogenously decreasing from 20 to 10 µmol m^-2^ s^-1^. The low-source: low-sink treatment also showed a homogeneous reduction of the curves’ asymptotes through the season but with lower values at the end of the season (6 µmol m^-2^ s^-1^). High-source treatments had the highest *A*_Nmax_, 24 µmol m^-2^ s^-1^, for the measurement taken at 309 GDD, and the lowest *A*_Nmax_, 5 µmol m^-2^ s^-1^, for measurement taken at 2500 and 2900 GDD. Interestingly, the decrease in *A*_Nmax_ was less homogeneous in these two treatments, and the curves split into two groups due to a strong decline of *A*_Nmax_ around 2000 GDD. This gap was greater when high-sink was combined with low-sources.

**Figure 4:**
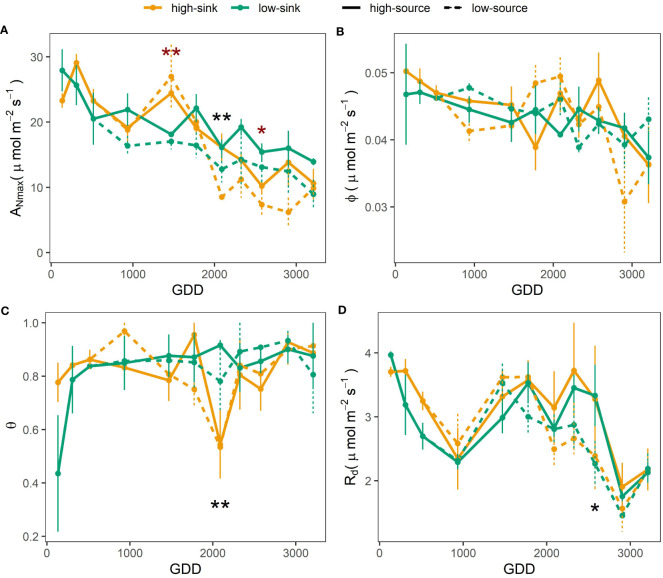
Effect of source: sink ratio on the light-saturated net photosynthetic rate (*A*_Nmax_, panel **(A)**, the quantum yield of assimilation (ϕ, panel **B)**, the curvature convexity factor (θ, panel **C)**, and the dark respiration rate (*R*d, panel **D)** of pistachio leaves as a function of leaf development expressed as growing degree days (GDD). Red asterisks indicate a significant effect of the sink, and black asterisks indicate a significant effect of the source on the photosynthetic parameters per each measurement day based on a Tukey test, with one and two asterisks representing p ≤ 0.05 and p ≤ 0.01 respectively.

A three-way ANOVA was performed to a reduced dataset, with the measurements starting June 1^st^, and including the defoliation treatment effects applied at that time (**Table 4**). Results of a two-way ANOVA performed using only the sink treatment and the entire dataset starting in April showed the same significance of the three-way ANOVA reported in **Table 4** for the factors Sink and Leaf Age. The three-way ANOVA demonstrated that the sink and source levels did not interact to affect the photosynthetic parameters monitored. The effect of leaf age on photosynthetic parameters was consistently highly significant (**Table 4**). Maximum photosynthetic rate (A_Nmax_) decreased through the season as leaf aged, attaining 50% lower values in fall with respect to spring (~ 10 µmol m^-2^ s^-1^ versus 20-25 µmol m^-2^ s^-1^, respectively) (**Figure 4A**). The quantum yield of assimilation (ϕ) was constant through the season with average values of 0.04 µmol m^-2^ s^-1^ (**Table 5**), and only very late in the season (after 2600 GDD) ϕ values were slightly lower (**Figure 4B**). The curvature convexity factor (θ) values ranged between 0.7 and 0.9 and showed a poor seasonal pattern (**Figure 4C**). Dark respiration (Rd) highest rates of 3.7 µmol m^-2^ s^-1^ were recorded earlier in the season, until 309 GDD, then values decreased constantly by 40%, to 2.3 µmol m^-2^ s^-1^ observed at 934 GDD. Rd rate increased again at 1500 GDD and then was reduced to minimum values at 2900 GDD (**Figure 4D**). Source manipulation influenced A_Nmax_ and R_d_ independently from leaf age (**Table 4**), with 20% higher A_Nmax_ values in the high-source than in the low-source branches (averages of 16.9 and 13.7 µmol m^-2^ s^-1^, respectively) and 10% lower R_d_ in the low-source branches with respect to the high-source branches (averages of 2.6 and 2.8 µmol m^-2^ s^-1^) (**Table 5**). The most significant impact of branch source on *A*_Nmax_ was observed at 2100 GDD (**Figure 4A**), when *A*_Nmax_ was 40% higher in high-source branches (**Figure 4A**). The most significant impact of branch source on R_d_ was observed later in the season, at 2800 GDD, when R_d_ was 25% higher in high-source branches (**Figure 4D**). The branch sink influenced A_Nmax_ and θ only in interaction with leaf age (**Table 4**). Specifically, high-sink branches had 30% higher *A*_Nmax_ at ~1500 GDD, but then 30% lower *A*_Nmax_ at 2500 GDD than low sink branches (**Figure 4A**) and θ was 40% higher in high-sink branches versus low-sink branches only at 2200 GDD (**Figure 4C**).

**Table 4:** Analysis of variance (ANOVA) testing the effects of source:sink manipulation and leaf age (expressed as accumulated growing degree days) on the photosynthetic parameters extrapolated from the light response curves.

Factors	A_Nmax_	φ	Θ	R_d_
Sink	ns	ns	ns	ns
Source	***	ns	ns	*
Leaf Age	***	**	*	***
Sink x Source	ns	ns	ns	ns
Sink x Leaf age	**	ns	*	ns
Source x Leaf age	ns	ns	ns	ns
Sink x Source x Leaf age	ns	ns	ns	ns

ns, not significant.

* p ≤ 0.05.

** p ≤ 0.01.

*** p ≤ 0.001.

**Table 5:** Seasonal average values (± error standard) of the different photosynthetic parameters extrapolated from the light response curves.

Source Treatment	A_Nmax_	φ	Θ	R_d_
High	19.1 ± 0.7 *^a^*	0.044 ± 0.001 *^ns^*	0.82 ± 0.02 *^ns^*	3.01 ± 0.10 *^a^*
Low	16.8 ± 1.0 *^b^*	0.044 ± 0.001	0.82 ± 0.02	2.82 ± 0.10 *^b^*
Sink Treatment				
High	17.6 ± 0.9 *ns*	0.044 ± 0.001 *ns*	0.82 ± 0.02 *ns*	3.00 ± 0.11 *ns*
Low	18.3 ± 0.8	0.044 ± 0.001	0.82 ± 0.02	2.84 ± 0.09

ns, non significant.For each parameter, different letters indicate significant differences due to a source effect (superscript) or sink effect (no superscript) based on a Tukey test (p ≤ 0.05).

**Figure 5:**
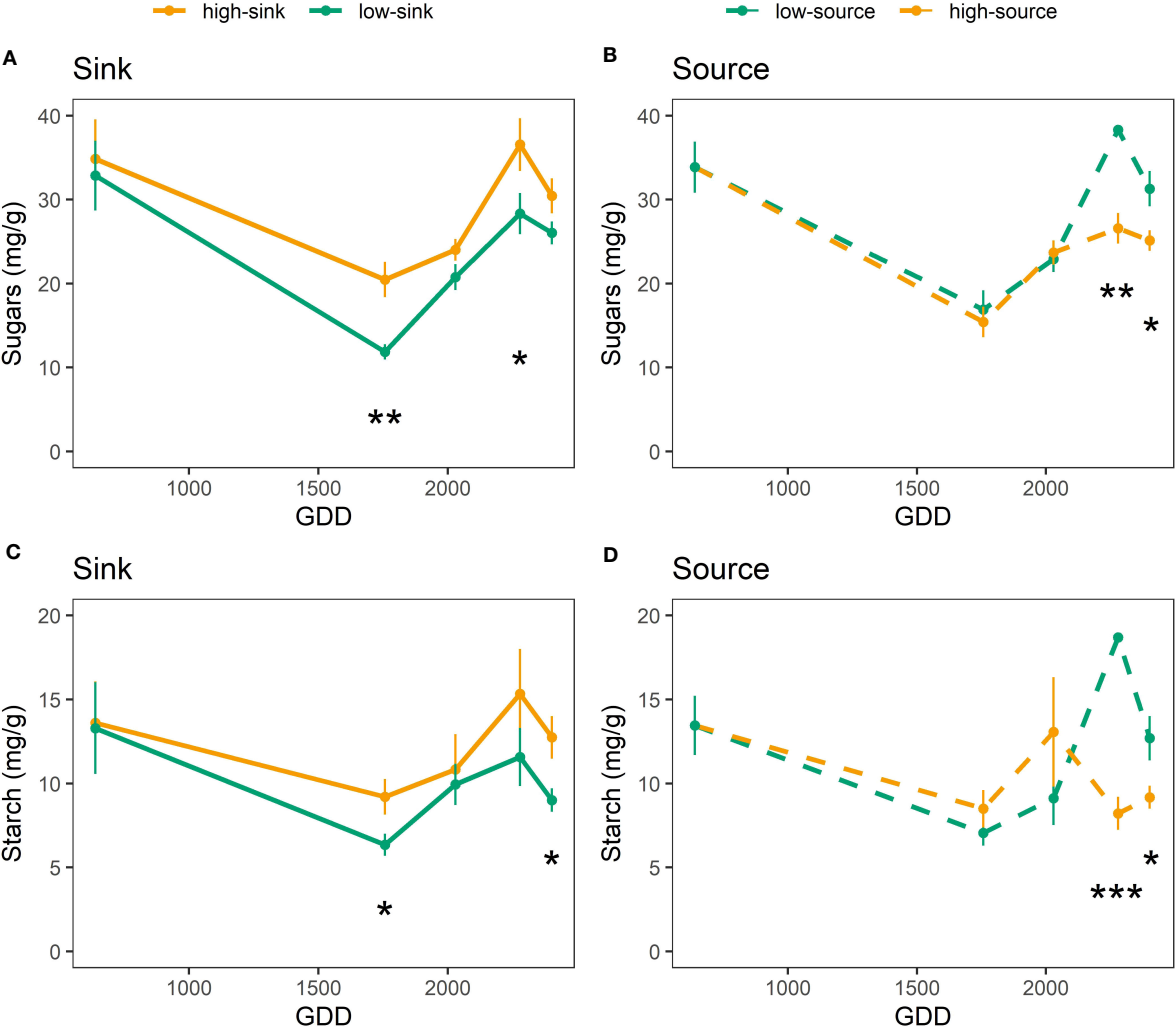
Effect of source and sink treatments on the sugar **(A, B)** and starch **(C, D)** content (mg/g) of pistachio leaves as a function of leaf age expressed as growing degree days. Black asterisks indicate a significant effect of the treatment per each day of measurement based on a Tukey test, with one, two and three asterisks representing p ≤ 0.05, p ≤ 0.01, and p ≤ 0.001, respectively.

**Figure 6:**
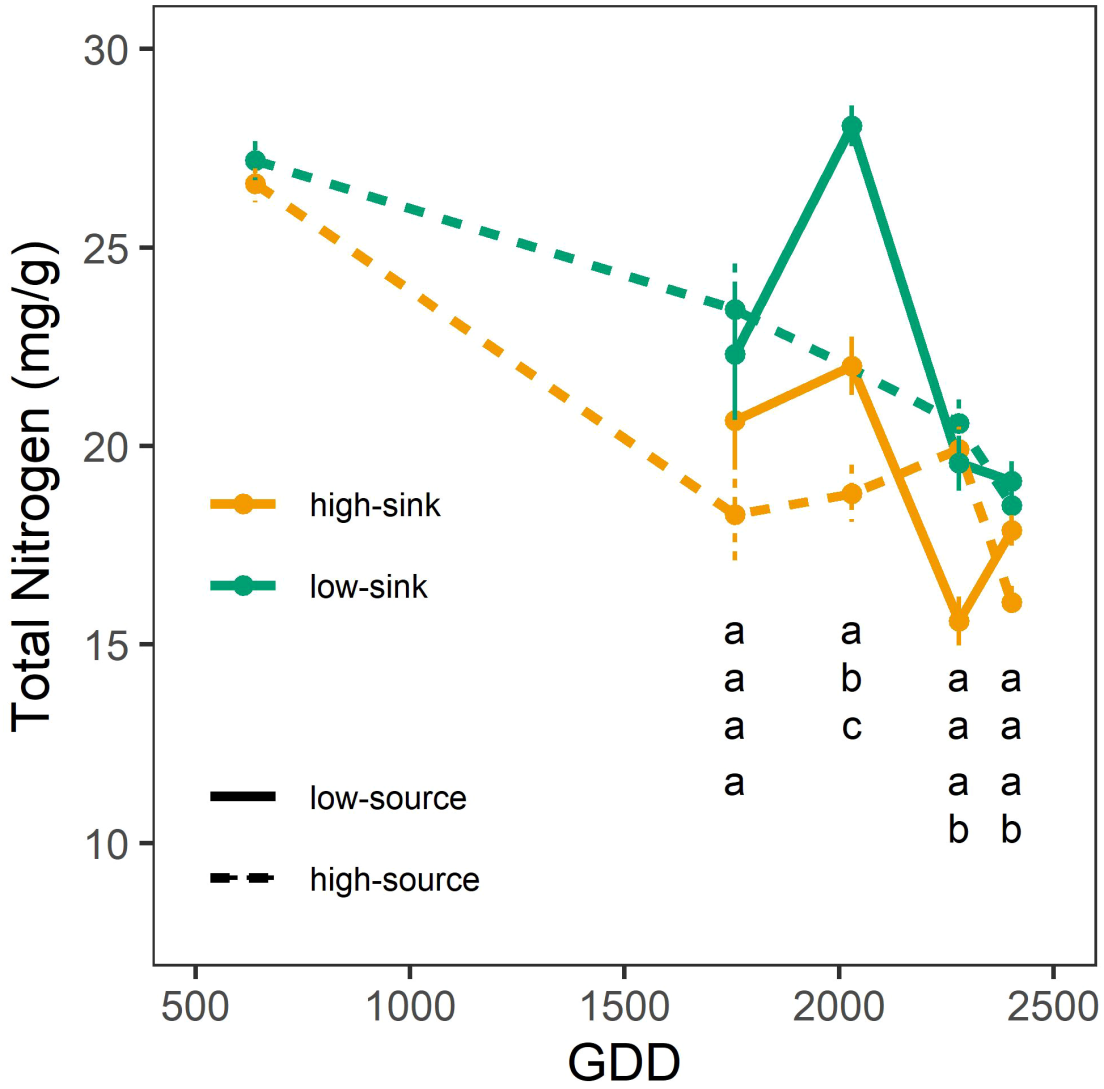
Effect of source and sink treatments on the nitrogen content (mg/g) of pistachio leaves as a function of leaf age expressed as growing degree days. Different letters indicate a significant effect of the combination of sink and source treatments per each day of measurement based on a Tukey test (p ≤ 0.05).

Both branch sink and source influenced leaf NSC content (**Table 6**), with branches with high sink and low source having 20-25% more sugars (30 mg g^-1^) and starch (13 mg g^-1^) than branches with low sink and high source (24 mg g^-1^ of sugars and 9 mg g^-1^ of starch) (**Figure 5**). Only the sink influenced leaf nitrogen content (**Table 6**), and high-sink branches had 25% less nitrogen than low-sink branches (17 vs 20 mg g^-1^, respectively) (**Figure 6**). No interaction between the two treatments was observed on any of the measured parameters that, nevertheless, varied with leaf age (**Table 6**) Interestingly, the effect of the source on leaf nitrogen and starch contents showed a significant interaction with leaf age (**Table 6**). Specifically, between 2000 and 2500 GDD, a strong decline in leaf nitrogen content paired with an increase in leaf starch content was observed in low-source branches (**Figures 5**, **6**). For leaf nitrogen content, also the sink showed an interaction with leaf age (**Table 6**). Differences in leaf nitrogen content were greater around 2000 GDD, when low-sink branches had 30% more nitrogen than high-sink branches, and decreased as time progressed, with low-sink branches having only 15% more nitrogen at 2500 GDD (**Figure 6**).

**Table 6:** Analysis of variance (ANOVA) testing the effects of fruit thinning, defoliation, and leaf age expressed as growing degree days on the leaf total nitrogen, sugar and starch content.

Factor	Total N	Sugar	Starch
Sink	***	***	**
Source	ns	***	***
Leaf age	***	***	*
Sink x Source	ns	ns	ns
Sink x Leaf age	**	ns	ns
Source x Leaf Age	***	ns	***
Sink x Source x Leaf age	**	ns	ns

ns, not significant.

* p ≤ 0.05.

** p ≤ 0.01.

*** p ≤ 0.001.

## 4 Discussion

Plant growth, reproduction and survival depend on their leaves’ ability to maximize carbon gain through the season. Hence, understanding the processes that impair or enhance leaf photosynthesis is essential to improve agroecosystems’ performance under variable conditions. As seen in **Figure 3** and reported for other tree crop species, leaf maximum photosynthetic rates (A_Nmax_) decreased through the season as the leaves aged (Jurik, 1986; Reich et al., 1991; Stirling et al., 1994; Warren and Adams, 2001; Warren, 2006). Interestingly, branch source-sink ratio affected the relation between A_Nmax_ and leaf age. The A_Nmax_ seasonal decline was gradual only in branches with a high source-sink ratio (**Figures 3B, D**). In branches with a low source-sink ratio, A_Nmax_ dropped abruptly by 70%, from 25 to 8 μmol m^-1^ s^-1^ in a two-week period, between 1700 and 2000 GDD. This data is also in line with previous studies on seasonal photosynthetic changes in pistachio reporting a reduction in A_Nmax_ at the beginning of July (Vemmos, 1994), especially pronounced in bearing branches and trees.

Two important events framed this quick decline in A_Nmax_. The first was at 1500 GDD, when the A_Nmax_ of the high-sink branches increased by 28% with respect to the previous measurement (**Figure 4**). This increase was not observed in the low-sink branches, which were assimilating significantly less carbon than the high-sink branches (18 μmol m^-1^ s^-1^ vs. 25 μmol m^-1^ s^-1^). Higher photosynthetic rates in branches with higher fruit loads are commonly reported in the literature (Iglesias et al., 2002; Andrade et al., 2019). Mid-July (~1.500 GDD) in pistachio is a period of intense carbon demand since the kernels are rapidly growing (Marino et al., 2022). Hence, the high sink strength for carbon exerted by the fruits in the highly loaded branches leads to leaf NSC depletion and eventually the upregulation of photosynthesis. The lack of differences in A_Nmax_ among treatments before 1500 GDD suggests that the high-sink branches, with three clusters, were not source-limited until the onset of kernel growth, but also that the low-sink branches, with a single fruit cluster, were not sink-limited. However, based on our recently published carbon budget model for pistachio (Marino et al., 2022), the branch photosynthetic apparatus at that time assimilates four times what is needed to support one single cluster with 16 nuts. Therefore, we hypothesize that A_Nmax_ was not inhibited before 1500 GDD in low-sink branches as NSC are not hyperaccumulating in leaves (**Figure 5**) but are being translocated to stronger sinks, such as heavily loaded branches and storage, that get depleted in the first part of the season (Tixier et al., 2020). This observation suggests that species with strong fruit sinks, such as pistachio, have seasonal changes in sink strength exerted by the growing fruits, which greatly affect the tree’s physiological responses to treatments and, potentially, management strategies. This characteristic may explain the unique behavior of this species observed in some irrigation studies where assimilation rates differ markedly for similar values of stomatal conductance rates depending on the fruit phenological stage (Gijon et al., 2011) and load (Marino et al., 2018a). In research studies, sampling and measurements should be replicated in bearing and non-bearing branches, or the bearing status of the branch or tree should be reported and considered when interpreting the results. In terms of orchard management, the data from this study can be implemented in carbon budget models and the results used to develop improved practices that consider tree crop load and its effect on leaf photosynthetic performance. The second significant impact of branch source-sink ratio on leaf photosynthesis was observed late in the growing season, at 2200 GDD (**Figure 3**). As described above, A_Nmax_ started to decline after 1500 GDD, more rapidly in branches with a low source-sink ratio (**Figure 3**). At 2200 GDD, the low-source branches had significantly lower A_Nmax_ than the high-source branches (9-13 μmol m^-1^ s^-1^ vs. 16 μmol m^-1^ s^-1^, respectively), and at 2600 GDD, the high-sink branches had lower A_Nmax_ than the low-sink ones. In both cases, the lower A_Nmax_ in branches with low source-sink ratios was associated with higher leaf NSC concentration (**Figure 5**). These results differ from studies showing that source-limited conditions produced by defoliation are generally associated with low levels of NSC and an increase in A_Nmax_ (Iglesias et al., 2002; Pinkard et al., 2011), and sink-limited conditions created by fruit thinning are generally associated with high leaf NSC concentration and lower A_Nmax_ (Iglesias et al., 2002; Andrade et al., 2019). Our data suggest that the substantial decline in A_Nmax_ observed at the end of the season could be the indirect result of branch source:sink ratio on leaf senescence. Specifically, the lowest branch source:sink ratio precipitated leaf senescence. The reduction of the curvature factor (θ) in highly loaded branches observed exactly at this date (**Figure 4**) support this theory, since lower θ has been associated with lower chlorophyll content (Leverenz, 1987; Leverenz, 1988), which in turn is a main symptom of leaf senescence, and correspond to a period when large differences in leaf nitrogen content were observed between high-sink and low-sink treatments (**Figure 6**).

Lv et al. (2020) reported similar results in wheat with increased reactive oxygen species production and degradation of chlorophyll-protein complexes and carbohydrates. Abeledo et al. (2020) found defoliation and non-thinning anticipated senescence in corn, but the response was affected by genotype and environmental conditions. This point could explain the different trend observed by other authors (e.g., Kumar et al., 2019 and Sekhon et al., 2012), who found anticipated leaf senescence induced by the lack of sinks. However, the consensus in the literature is that NSC hyperaccumulation in the source (leaf) tissue was associated with premature senescence (Christensen et  al, 1981; Ceppi et al., 1987; Sekhon et al., 2012), consistent with what we observed in our study for the lowest source:sink ratio treatment (**Figure 5**).

It is beyond the scope of this study to elucidate the mechanisms associated with leaf senescence in response to source: sink balance. However, an interesting and unexpected result was that the interaction of the two treatments (fruit thinning and defoliation) on photosynthesis was never significant, potentially suggesting a different response mechanism of pistachio to sink-limited and source-limited conditions. This different mechanism was indicated by N content changes. Declining A_Nmax_ in high-sink branches was accompanied by a clear reduction in leaf N content during senescence in early August (**Figure 6**). However, defoliation did not significantly affect leaf N levels. This result suggests N remobilization to the growing kernels could be a major factor in reducing photosynthesis in heavily loaded branches at the end of the season (Mei and Thimann, 1984; Morris and Pauisen, 1985). In addition, defoliation but not fruit thinning affected leaf dark respiration (R_d_) rates, with leaves from low-source branches having significantly lower R_d_, especially toward the end of the season (**Table 3** and **Figure 4B**). Earlier studies on the impact of defoliation on respiration rates show contrasting results, with both increases and decreases being reported (Ovaska et al., 1993; Reich et al., 1993; Layne and Flore, 1995; Pinkard et al., 2007). In our case, the decline in R_d_ under defoliation corresponded perfectly to the decline in A_Nmax_ and to the NSC accumulation in leaves. A parallel decrease between A_Nmax_ and R_d_ is commonly observed since the two parameters may be affected by similar factors (Amthor, 2012) and may suggest a decrease in biosynthesis and prioritization of assimilate storage. Storage implies lower respiratory costs than biosynthesis (Amthor, 2012). On the contrary, non-defoliated branches showed a respiration peak prior to R_d_ decline, a typical symptom of senescence onset and generally associated with an increase in metabolic rate to support the energetic needs of remobilization.

## 5 Conclusion

This study demonstrates that the source-sink balance at the branch and, potentially, tree level influences the photosynthetic capacity of individual pistachio leaves. An unequal source-sink ratio can promote higher photosynthesis during period of high sink activity, but also accelerate leaf senescence, precipitating a decline in photosynthetic activity late in the growing season. Fall assimilation is important for deciduous species, and increased leaf lifespan can enhance reserve accumulation to support winter dormancy and the following year’s bloom. Based on these results, heavily fruiting branches may have impaired photosynthetic capacities starting in middle-late August. Management could be optimized based on tree crop load. Specifically, in years with high crop load, thinning strategies could be used to improve multi-year tree carbon balance and productivity, and irrigation reductions or cut-offs during post-harvest could be implemented without significantly impacting tree carbon resources. Also, the timing for zinc fall applications could be selected considering also tree crop load and associated leaf photosynthetic and translocation potential. Finally, breeding programs could benefit from the results of this study. If stay-green traits in high-yielding branches or trees are associated with lower alternate bearing, a highly desired trait for growers, they could be used for cultivar selection and in crosses.

Our data suggest that studies focusing on photosynthetic capacity or primary gross production of tree crop species with strong alternate bearing behaviors, such as *Pistacia* spp., should consider the physiological changes the trees undergo during high bearing and low bearing years. Data from this study can be potentially implemented in available carbon budget models to facilitate the adaptation of management practices to tree sink-source balance.

## Data availability statement

The raw data supporting the conclusions of this article will be made available by the authors, without undue reservation.

## Author contributions

GM, PG-D and BB-U conceived the experiment; GM, PG-D, ES, JA, BB-U set up the experiment and collected the data. GM, PG-D and EF-S analysed the data. All authors contributed to data interpretation. GM and PG-D wrote the manuscript. All authors contributed to the article and approved the submitted version.
